# *Vital Signs:* HIV Infection, Diagnosis, Treatment, and Prevention Among Gay, Bisexual, and Other Men Who Have Sex with Men — United States, 2010–2019

**DOI:** 10.15585/mmwr.mm7048e1

**Published:** 2021-12-03

**Authors:** Marc A. Pitasi, Linda Beer, Susan Cha, Shacara Johnson Lyons, Angela L. Hernandez, Joseph Prejean, Linda A. Valleroy, Stacy M. Crim, Lindsay Trujillo, Dominique Hardman, Elizabeth M. Painter, Jacqueline Petty, Jonathan H. Mermin, Demetre C. Daskalakis, H. Irene Hall

**Affiliations:** ^1^Division of HIV Prevention, National Center for HIV, Viral Hepatitis, STD, and TB Prevention, CDC; ^2^ICF International, Fairfax, Virginia; ^3^Office of the Director, National Center for HIV, Viral Hepatitis, STD, and TB Prevention, CDC.

## Abstract

**Background:**

Men who have sex with men (MSM) accounted for two thirds of new HIV infections in the United States in 2019 despite representing approximately 2% of the adult population.

**Methods:**

CDC analyzed surveillance data to determine trends in estimated new HIV infections and to assess measures of undiagnosed infection and HIV prevention and treatment services including HIV testing, preexposure prophylaxis (PrEP) use, antiretroviral therapy (ART) adherence, and viral suppression, as well as HIV-related stigma.

**Results:**

The estimated number of new HIV infections among MSM was 25,100 in 2010 and 23,100 in 2019. New infections decreased significantly among White MSM but did not decrease among Black or African American (Black) MSM and Hispanic/Latino MSM. New infections increased among MSM aged 25–34 years. During 2019, approximately 83% of Black MSM and 80% of Hispanic/Latino MSM compared with 90% of White MSM with HIV had received an HIV diagnosis. The lowest percentage of diagnosed infection was among MSM aged 13–24 years (55%). Among MSM with a likely PrEP indication, discussions about PrEP with a provider and PrEP use were lower among Black MSM (47% and 27%, respectively) and Hispanic/Latino MSM (45% and 31%) than among White MSM (59% and 42%). Among MSM with an HIV diagnosis, adherence to ART and viral suppression were lower among Black MSM (48% and 62%, respectively) and Hispanic/Latino MSM (59% and 67%) compared with White MSM (64% and 74%). Experiences of HIV-related stigma among those with an HIV diagnosis were higher among Black MSM (median = 33; scale = 0–100) and Hispanic/Latino MSM (32) compared with White MSM (26). MSM aged 18–24 years had the lowest adherence to ART (45%) and the highest median stigma score (39).

**Conclusion:**

Improving access to and use of HIV services for MSM, especially Black MSM, Hispanic/Latino MSM, and younger MSM, and addressing social determinants of health, such as HIV-related stigma, that contribute to unequal outcomes will be essential to end the HIV epidemic in the United States.

## Introduction

Gay, bisexual, and other men who have sex with men (MSM) have been disproportionately affected by HIV since the onset of the epidemic and have been a priority population for HIV prevention and treatment (*1*). Despite focused prevention efforts, approximately two thirds of new HIV infections in the United States occur in MSM (*2*). Advances in HIV prevention and treatment have made HIV infection increasingly preventable, but new infections have continued. Preexposure prophylaxis (PrEP) is highly effective in preventing infection, and consistent antiretroviral therapy (ART) enables persons with HIV to become virally suppressed and prevents transmission to others (*3*,*4*). By maximizing these advances, the Ending the HIV Epidemic in the U.S. (EHE) initiative aims to reduce the number of new HIV infections in the United States by 90% by 2030; 57 state and local jurisdictions began implementing the initiative in 2020.* EHE goals cannot be achieved without substantial reductions in HIV infections among MSM. CDC analyzed data from three national surveillance systems to assess HIV prevention and treatment outcomes among MSM in the United States during the years before EHE implementation and the progress needed to reach EHE and other national goals (Supplementary Box, https://stacks.cdc.gov/view/cdc/111462).

## Methods

CDC assessed select outcomes related to the use of important HIV prevention services and steps in the HIV care continuum^†^ among MSM overall and by race/ethnicity and age group using data from the National HIV Surveillance System (NHSS), National HIV Behavioral Surveillance (NHBS), and Medical Monitoring Project (MMP). All methods are described elsewhere (Supplementary Appendix, https://stacks.cdc.gov/view/cdc/111463), including the outcomes and years of data analyzed. To assess changes in estimated HIV infections, the z-test was used to compare changes from 2010 to 2019; p-values <0.05 indicated statistically significant change. Estimates from MMP were weighted to represent the population of adults with diagnosed HIV infection in the United States. Unweighted frequencies, weighted percentages, and 95% CIs were generated from NHBS and MMP data. Estimates with a denominator sample size <30 were not reported. All analyses were conducted using SAS software (version 9.4; SAS Institute). This activity was reviewed by CDC and was conducted consistent with applicable federal law and CDC policy.^§^

## Results

**Estimated number of new HIV infections and percentage of infections that were diagnosed.** Using NHSS data, the estimated number of new HIV infections among MSM was 25,100 in 2010 and 23,100 in 2019 (p = 0.05) (Figure). During this period, infections significantly decreased from 7,500 to 5,100 among White MSM (p<0.01) but did not decline significantly among Black or African American (Black) MSM (9,000 to 8,900; p = 0.90) and Hispanic/Latino MSM (6,800 to 7,900; p = 0.10). Infections decreased among MSM aged 13–24 years (10,400 to 5,700; p<0.01) and 45–54 years (2,900 to 2,000; p<0.01) but increased among MSM aged 25–34 years (6,700 to 10,000; p<0.01).

**FIGURE:**
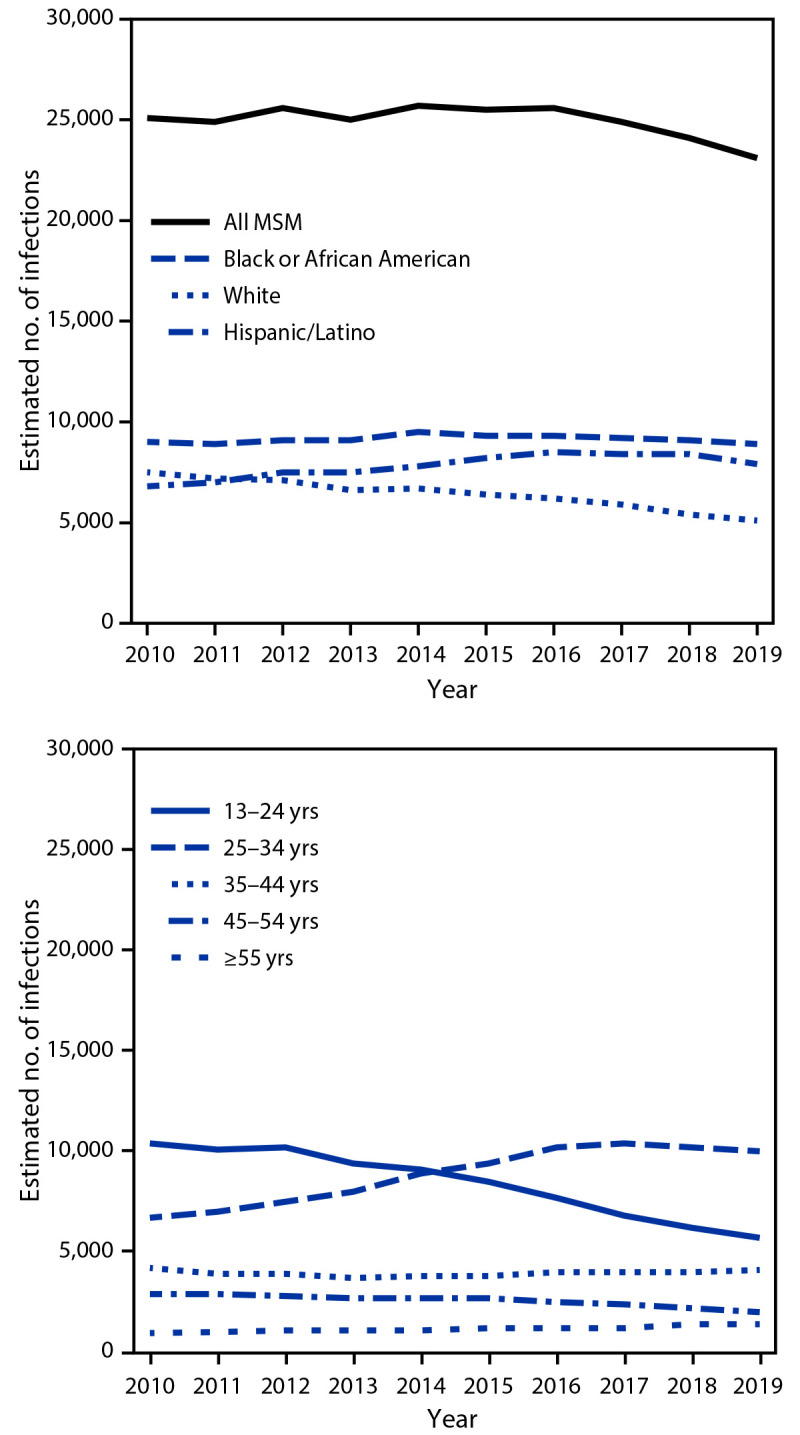
Estimated number of new HIV infections among gay, bisexual, and other men who have sex with men, by race/ethnicity and age category — United States, 2010–2019 **Abbreviation:** MSM = men who have sex with men.

Among the estimated 692,900 MSM living with HIV infection in 2019, 85% had received an HIV diagnosis (Table 1). A lower percentage of Black MSM (83%) and Hispanic/Latino MSM (80%) with HIV had received a diagnosis than did White MSM (90%). The lowest percentages of diagnosed infection were among MSM aged 13–24 years (55%) and 25–34 years (71%).

**TABLE 1:** Percentage of HIV infections diagnosed, percentage of persons receiving HIV testing in the past 12 months, and percentage of missed opportunities for HIV testing in the past 12 months among gay, bisexual, and other men who have sex with men, by race/ethnicity and age group — United States, 2017 and 2019

Characteristic	Diagnosed HIV infection (2019)*		Tested in past 12 mos (2017)^†^		Missed opportunity for testing in past 12 mos (2017)^†,§^	
	No.	% (95% CI)	No.	% (95% CI)	No.	% (95% CI)
**Total**	**692,900**	**84.8 (84.1–85.5)**	**7,577**	**78.6 (77.1–80.0)**	**1,181**	**77.8 (73.9–81.7)**
**Race/Ethnicity**						
American Indian/Alaska Native	—^¶^	—^¶^	53	69.0** (53.1–84.8)	—^††^	—^††^
Asian	—^¶^	—^¶^	194	81.0 (73.3–88.7)	35	88.6 (78.0–99.1)
Black/African American	219,200	82.6 (81.4–83.9)	1,965	80.7 (78.1–83.4)	283	73.6 (65.3–82.0)
Hispanic/Latino**^§§^**	186,800	80.3 (79.0–81.7)	2,098	77.3 (74.8–79.9)	315	75.6 (68.4–82.9)
Native Hawaiian/Other Pacific Islander	—^¶^	—^¶^	35	85.1** (65.7–100.0)	—^††^	—^††^
White	239,600	90.0 (88.7–91.3)	2,804	78.0 (75.5–80.5)	481	78.6 (72.5–84.7)
Multiple races^¶¶^	—^¶^	—^¶^	387	81.2 (75.2–87.2)	48	82.7** (64.9–100.0)
**Age group, yrs**						
13–24	37,100	55.1 (52.8–57.6)	—***	—***	—***	—***
18–24	—***	—***	1,359	79.7 (76.5–83.0)	197	77.0 (67.8–86.2)
25–34	164,600	71.2 (70.0–72.4)	3,266	81.3 (79.2–83.4)	372	80.9 (74.6–87.1)
35–44	135,200	84.0 (82.8–85.3)	1,397	79.3 (76.1–82.4)	200	74.7 (65.2–84.2)
45–54	155,300	92.4 (91.4–93.5)	999	71.8 (67.6–76.0)	220	71.9 (62.6–81.2)
≥55	200,600	96.1 (95.0–97.2)	556	65.0 (58.2–71.9)	192	82.5 (73.0–91.9)

* Based on data reported through December 2020 to the National HIV Surveillance System for year-end 2019. Percentages are estimated based on a CD4 depletion model. Defined as the number of persons who received an HIV diagnosis divided by the estimated number of persons with HIV (diagnosed and undiagnosed).

^†^ Based on data collected by National HIV Behavioral Surveillance in 2017 in 23 U.S. urban areas (Atlanta, Georgia; Baltimore, Maryland; Boston, Massachusetts; Chicago, Illinois; Dallas, Texas; Denver, Colorado; Detroit, Michigan; Houston, Texas; Los Angeles, California; Memphis, Tennessee; Miami, Florida; Nassau and Suffolk counties, New York; New Orleans, Louisiana; New York City, New York; Newark, New Jersey; Philadelphia, Pennsylvania; Portland, Oregon; San Diego, California; San Francisco, California; San Juan, Puerto Rico; Seattle, Washington; Virginia Beach, Virginia; and Washington, DC). Excludes persons who tested HIV-positive >12 months ago.

^§^ Defined as visiting a health care provider in the past 12 months without being offered an HIV test. Excludes persons who tested HIV-positive >12 months ago and who tested in the past 12 months.

^¶^ Estimates are not available because of high relative standard errors.

** Estimates have a CI width >30 and should be interpreted with caution.

^††^ Estimates are not available because denominator sample sizes are <30.

^§§^ Hispanic/Latino men who have sex with men could be any race.

^¶¶^ Represents persons identified as having multiple race categories selected.

*** National HIV Behavioral Surveillance did not collect data from persons aged 13–17 years. Data from the National HIV Surveillance System are presented for persons aged 13–24 years.

**Uses of and barriers to prevention and treatment services.** CDC examined NHBS data collected in 2017 among MSM who attended venues where the majority of attending men were MSM in 23 U.S. urban areas^¶^ and did not report a positive HIV test >12 months before the interview. Among these 7,577 MSM, 79% were tested for HIV in the past 12 months (Table 1). Among 1,181 MSM who visited a health care provider but had not tested in the past 12 months, 78% were not offered an HIV test. Neither HIV testing in the past 12 months nor having visited a provider without testing in the past 12 months differed by race/ethnicity. MSM aged 45–54 years and ≥55 years had the lowest percentages of testing in the past 12 months; visiting providers without a test did not differ by age group.

Approximately one half (52%) of HIV-negative MSM with likely PrEP indications** reported having discussed PrEP with a health care provider in the past 12 months, and approximately one third (36%) had used PrEP in the past 12 months (Table 2). Discussing and using PrEP were lowest among MSM aged 18–24 years (44% and 27%, respectively) and ≥55 years (46% and 24%), and varied by race/ethnicity (Black MSM [47% and 27%], Hispanic/Latino MSM [45% and 31%], and White MSM [59% and 42%]).

**TABLE 2:** Percentage of gay, bisexual, and other men who have sex with men with a likely indication for preexposure prophylaxis who discussed preexposure prophylaxis with a health care provider in the past 12 months or used preexposure prophylaxis in the past 12 months, by race/ethnicity and age group — United States, 2017

Characteristic	Discussed PrEP with health care provider in past 12 mos*		Used PrEP in past 12 mos*	
	No.	% (95% CI)	No.	% (95% CI)
**Total**	4,466	51.5 (49.1–53.9)	4,466	35.5 (33.0–38.0)
**Race/Ethnicity**				
American Indian/Alaska Native	—^¶^	—^¶^	—^¶^	—^¶^
Asian	111	61.1 (48.3–74.0)	111	47.4 (34.1–60.6)
Black/African American	962	47.2 (42.5–51.8)	962	27.2 (22.7–31.7)
Hispanic/Latino**^†^**	1,250	45.2 (41.2–49.2)	1,250	31.3 (27.5–35.2)
Native Hawaiian/Other Pacific Islander	—^¶^	—^¶^	—^¶^	—^¶^
White	1,841	58.5 (54.9–62.0)	1,841	42.2 (38.4–46.0)
Multiple races^§^	230	45.9 (36.0–55.7)	230	30.1 (21.5–38.7)
**Age group, yrs**				
18–24	837	43.6 (38.3–49.0)	837	26.7 (22.2–31.2)
25–34	2,073	52.6 (49.2–56.0)	2,073	36.8 (33.3–40.3)
35–44	845	59.9 (55.0–64.8)	845	44.7 (39.7–49.8)
45–54	480	48.8 (41.4–56.1)	480	35.7 (28.4–42.9)
≥55	231	46.4 (36.9–56.0)	231	23.7 (15.1–32.3)

**Abbreviations:** MSM = men who have sex with men; NHBS = National HIV Behavioral Surveillance; PrEP = preexposure prophylaxis.

* Based on data collected by NHBS in 2017 in 23 U.S. urban areas (Atlanta, Georgia; Baltimore, Maryland; Boston, Massachusetts; Chicago, Illinois; Dallas, Texas; Denver, Colorado; Detroit, Michigan; Houston, Texas; Los Angeles, California; Memphis, Tennessee; Miami, Florida; Nassau and Suffolk counties, New York; New Orleans, Louisiana; New York City, New York; Newark, New Jersey; Philadelphia, Pennsylvania; Portland, Oregon; San Diego, California; San Francisco, California; San Juan, Puerto Rico; Seattle, Washington; Virginia Beach, Virginia; and Washington, DC). Restricted to MSM with likely clinical indications for PrEP, who had a negative NHBS HIV test result after the NHBS interview, had a male sex partner who was HIV-positive or two or more male sex partners in the past 12 months, and had condomless anal sex with a male sex partner or a sexually transmitted infection (i.e., syphilis, gonorrhea, or chlamydia) in the past 12 months.

^†^ Hispanic/Latino MSM could be any race.

^§^ Represents persons identified as having multiple race categories selected.

^¶^ Estimates not available because denominator sample sizes are <30.

Using MMP data collected during June 2018–May 2019 among MSM with diagnosed HIV infection, an estimated 58% were fully ART dose-adherent in the past 30 days (Table 3). Adherence was lowest among MSM aged 18–24 years (45%) and 25–34 years (48%) and Black MSM (48% compared with 64% among White MSM). Overall, 68% of MSM with diagnosed HIV infection were virally suppressed. Black MSM (62%), American Indian or Alaska Native MSM (65%), and MSM aged 25–34 years (65%) had the lowest percentages of viral suppression.

**TABLE 3:** Among gay, bisexual, and other men who have sex with men with diagnosed HIV infection, percentage with antiretroviral therapy adherence, percentage with viral suppression, and median HIV-related stigma scores, by race/ethnicity and age group — United States, 2018 and 2019

Characteristic	ART adherence (2018)*		Viral suppression (2019)^†^		HIV-related stigma score (2018)^§^	
	No.	% (95% CI)	No.	%	No.	Median (95% CI)
**Total**	1,869	58.3 (54.9–61.7)	528,606	68.1	1,873	29.3 (28.0–30.5)
**Race/Ethnicity**						
American Indian/Alaska Native	—^¶^	—^¶^	1,538	64.7	—^¶^	—^¶^
Asian	—^¶^	—^¶^	9,779	71.7	—^¶^	—^¶^
Black/African American	503	48.3 (40.2–56.3)	161,072	61.6	528	32.8 (29.3–36.3)
Hispanic/Latino**	440	58.7 (53.4–64.1)	135,301	66.6	436	32.0 (29.6–34.3)
Native Hawaiian/Other Pacific Islander	—^¶^	—^¶^	590	66.2	—^¶^	—^¶^
White	784	64.1 (59.4–68.9)	195,335	73.5	770	26.1 (24.0–28.2)
Multiple races^††^	104	55.7 (44.8–66.7)	24,643	74.5	103	30.4 (24.2–36.6)
American Indian/Alaska Native, Asian, or Native Hawaiian/Other Pacific Islander^§§^	38	60.2^¶¶^ (40.2–80.2)	—^§§^	—^§§^	36	20.3 (12.0–28.7)
**Age group, yrs**						
13–24	—***	—***	19,520	66.2	—***	—***
18–24	53	44.6^¶¶^ (29.5–59.6)	—***	—***	56	39.3 (30.0–48.7)
25–34	319	47.7 (39.7–55.7)	105,957	65.0	332	33.6 (30.6–36.6)
35–44	346	53.7 (47.5–59.9)	101,620	66.1	353	31.5 (29.7–33.4)
45–54	523	55.7 (50.3–61.1)	140,157	69.3	517	28.7 (26.8–30.6)
≥55	628	69.6 (64.9–74.4)	161,352	70.6	615	25.4 (23.5–27.3)

**Abbreviations:** ART = antiretroviral therapy; MSM = men who have sex with men; NHBS = National HIV Behavioral Surveillance; PrEP = preexposure prophylaxis.

* Based on data collected by the Medical Monitoring Project during June 2018–May 2019. ART adherence was defined as taking 100% of ART doses in the past 30 days among MSM currently taking ART.

^†^ Based on data reported through December 2020 to the National HIV Surveillance System for year-end 2019. Viral suppression was defined as the number of MSM with a viral load test result of <200 copies of HIV RNA per mL at last test divided by the number of MSM with diagnosed HIV infection.

^§^ Based on data collected by the Medical Monitoring Project during June 2018–May 2019. HIV-related stigma was measured using a 10-item scale that measures four dimensions of HIV stigma: personalized stigma during the past 12 months, current disclosure concerns, current negative self-image, and current perceived public attitudes about persons with HIV. The stigma score ranged from 0 to 100, with 0 indicating no stigma and 100 indicating highest stigma. A median score was calculated based on responses on a five-point Likert scale to each item. Median scores with nonoverlapping 95% CIs were considered to be meaningfully different. Median scores were interpreted in the context of the national goal of reducing HIV-related stigma by 2025 by at least 50% from the 2018 baseline median score of 31.

^¶^ Estimates are not available because denominator sample sizes are <30.

** Hispanic/Latino MSM could be any race.

^††^ Represents persons identified as having multiple race categories selected.

^§§^ Viral suppression percentages are presented separately for American Indian or Alaska Native persons, Asian persons, and Native Hawaiian or Other Pacific Islander persons.

^¶¶^ Estimates have a CI width >30 and should be interpreted with caution.

*** The Medical Monitoring Project did not collect data from persons aged 13–17 years. Data from the National HIV Surveillance System are presented for persons aged 13–24 years.

The median HIV-related stigma score^††^ among MSM with diagnosed HIV infection was 29 on a scale of 0 to 100. MSM aged 18–24 years had the highest median score (39). Black MSM (33) and Hispanic/Latino MSM (32) had higher median scores than did White MSM (26).

## Discussion

These findings indicate that new HIV infections among Black MSM and Hispanic/Latino MSM did not decrease during the decade before EHE implementation despite decreases or stable numbers among other MSM subgroups, and new infections increased among MSM aged 25–34 years. Use of many prevention and treatment strategies were less prevalent among Black MSM, Hispanic/Latino MSM, and younger MSM. Longstanding inequities in access to and delivery of needed services among some racial/ethnic and age groups, particularly Black MSM and Hispanic/Latino MSM, have persisted despite focused efforts to prevent HIV in these populations for decades. Efforts to reduce these and other disparities must address their root causes, including systemic racism, stigma, discrimination, homophobia, poverty, homelessness, and unequal access to care and prevention services (*1*).

Achieving the EHE goals to reduce the number of HIV infections by 90% by 2030 will require that at least 95% of infections are diagnosed and 95% of persons with diagnosed HIV infection are virally suppressed (Supplementary Box, https://stacks.cdc.gov/view/cdc/111462); the most recent available data indicate that among MSM, only 85% of HIV infections are diagnosed and 68% of MSM with diagnosed HIV infection are virally suppressed. Approximately 20% of MSM not previously receiving a diagnosis of HIV infection had not been tested for HIV in the past year, which is inconsistent with CDC recommendations that all sexually active MSM be tested at least annually (*5*). Missed clinical opportunities for testing were common among MSM who had not been tested in the past year. Further, PrEP was used by only one third of MSM for whom it was likely indicated, well below the EHE target of 50% PrEP coverage (Supplementary Box, https://stacks.cdc.gov/view/cdc/111462). Median HIV-related stigma scores were nearly double the national target (*6*). The persistence of HIV-related stigma might hinder access to testing, prevention, and treatment for MSM, thus potentially undermining progress toward national goals. Together, these findings suggest the need for innovative approaches that can better deliver testing, prevention, and treatment services to MSM.

Several innovative and culturally appropriate strategies have successfully reduced barriers to access of services and might help achieve national goals of improving prevention, diagnosis, and treatment of HIV infection among MSM.^§§^ For example, HIV testing scale-up has been determined to be cost-effective across diverse local conditions (*7*). Some jurisdictions have successfully implemented programs that increased screening frequency among MSM (*8*). Numerous strategies have been implemented to deliver HIV testing services to MSM by expanding or tailoring existing clinical screening programs, enhancing community-based testing options, or providing HIV self-tests (*9*). HIV self-testing can be a cost-saving delivery strategy (*10*) with potential to mitigate HIV-related stigma and better reach MSM (*11*). Multiple jurisdictions have demonstrated that HIV self-test distribution programs can successfully deliver HIV testing to racial/ethnic minority MSM and MSM not reached by other testing programs (*12*). CDC recently supported a national self-test distribution program designed to improve access to HIV testing for those who had not been previously reached.^¶¶^

To improve HIV care outcomes, strategies and approaches supported by the Ryan White HIV/AIDS Program (RWHAP) can be scaled up to reach all U.S. facilities that provide HIV care. RWHAP-funded facilities deliver comprehensive care and essential support services to approximately one half of persons with diagnosed HIV infection in the United States through enhanced collaboration with local partners, community engagement, effective data collection, and provider training. RWHAP activities have led to recent increases in viral suppression among MSM from 84.7% in 2015 to 89.1% in 2019 (*13*). These activities also reduced racial/ethnic disparities by as much as one third by addressing structural factors, such as unstable housing, that impede access to HIV care and treatment (*14*). Other programs have used surveillance data to identify persons not receiving care and have successfully engaged them using interventions such as patient navigation to reduce barriers to access (*15*).

Prevention of new infections can be enhanced by ensuring that PrEP providers are available in communities most affected by HIV and by integrating PrEP services into existing clinical settings, such as sexually transmitted disease (STD) clinics. As part of the EHE initiative, CDC supports local efforts to build the capacity of STD clinics to implement innovative, locally tailored strategies to provide PrEP and other HIV prevention services to MSM at risk for acquiring HIV.*** Such clinics often function as safety nets for populations with limited access to other sources of care, thus providing crucial prevention services to underserved populations and reducing racial/ethnic disparities in care (*16*). Local programs have highlighted opportunities to improve rapid PrEP initiation and navigation services for STD clinic patients with ongoing risk for HIV infection (*17*).

Emerging interventions and delivery strategies for testing, prevention, and treatment might also reduce barriers to accessing services and reaching EHE goals. Telehealth and other novel care models can provide additional options for accessing and improving adherence to HIV treatment and PrEP (*18**,**19*). Development of long-acting HIV medications could further expand access and facilitate adherence to PrEP and ART (*20*). Such innovative interventions and delivery strategies should be prioritized for use among the most disproportionately affected groups, including Black and Hispanic/Latino MSM and younger MSM. Their implementation should be designed to address structural factors that often limit access to and use of these technologies. To further promote engagement in HIV services and reduce HIV-related stigma, MSM should be engaged in HIV prevention or treatment services regardless of their HIV status (i.e., a status neutral approach) (*1*). This approach helps persons with HIV and persons at higher risk for infection receive the services needed to prevent HIV transmission or acquisition without status-specific structures that reinforce stigma and other related barriers.

The findings in this report are subject to at least seven limitations. First, data were collected before the onset of the COVID-19 pandemic and do not reflect disruptions in HIV testing, prevention, or treatment services. Second, MMP and NHBS data were self-reported and are subject to recall and social desirability biases. Third, NHBS behavioral measures of likely PrEP indication did not correspond directly with clinical guidelines and might have underestimated MSM with likely PrEP indications who discussed PrEP with a health care provider or used PrEP. Fourth, viral suppression measures presented here did not include data from jurisdictions without complete laboratory reporting and therefore might not be representative of all persons with diagnosed HIV infection in the United States. Fifth, the small number of MSM in some subgroups might have reduced the reliability of their estimates. Sixth, outcomes based on NHSS data for MSM aged 13–24 years were presented for a single age category, potentially obscuring differences in this developmentally diverse group. Finally, NHSS data presented by transmission category (i.e., male-to-male sexual contact) are based on sex at birth. Therefore, estimates based on NHSS data included some persons with a gender identity other than male (e.g., transgender women) who were classified as MSM based on their sex at birth.

Intensified and innovative efforts to expand access to HIV testing, prevention, and treatment services for MSM, particularly Black MSM, Hispanic/Latino MSM, and younger MSM, are required to decrease health disparities and reduce new HIV infections by 90% to reach EHE goals. Jurisdictions should identify and implement those programs and interventions most responsive to local needs and acceptable to disproportionately affected populations of MSM. All programs should implement a status neutral approach to reduce barriers to prevention, testing, and treatment by breaking down institutional barriers and reducing HIV-related stigma.

**Summary**

**What is already known about this topic?**

Gay, bisexual, and other men who have sex with men (MSM) are disproportionately affected by HIV.

**What is added by this report?**

This analysis of national surveillance data found that the estimated number of new HIV infections among MSM did not change overall during 2010–2019; infections decreased among White MSM but not among Black MSM or Hispanic/Latino MSM. Most measures of use of HIV prevention and treatment services were lower among Black MSM and Hispanic/Latino MSM than White MSM and younger MSM compared with other age groups.

**What are the implications for public health practice?**

Improving access to and use of HIV services for MSM, particularly Black MSM, Hispanic/Latino MSM, and younger MSM, is essential to ending the HIV epidemic in the United States.

## Footnotes

*
https://www.hiv.gov/federal-response/ending-the-hiv-epidemic/overview

†
https://www.cdc.gov/hiv/pdf/library/factsheets/cdc-hiv-care-continuum.pdf

§ 45 C.F.R. part 46.102(l)(2), 21 C.F.R. part 56; 42 U.S.C. Sect. 241(d); 5 U.S.C. Sect. 552a; 44 U.S.C. Sect. 3501 et seq.

¶ Atlanta, Georgia; Baltimore, Maryland; Boston, Massachusetts; Chicago, Illinois; Dallas, Texas; Denver, Colorado; Detroit, Michigan; Houston, Texas; Los Angeles, California; Memphis, Tennessee; Miami, Florida; Nassau and Suffolk counties, New York; New Orleans, Louisiana; New York City, New York; Newark, New Jersey; Philadelphia, Pennsylvania; Portland, Oregon; San Diego, California; San Francisco, California; San Juan, Puerto Rico; Seattle, Washington; Virginia Beach, Virginia; and Washington, DC.

** MSM likely indicated for PrEP included those who 1) received a negative NHBS HIV test result after the NHBS interview; 2) had two or more male sex partners or any male sex partner with HIV infection within the past 12 months; and 3) engaged in condomless anal sex or had a bacterial sexually transmitted infection within the past 12 months.

†† Participants indicated their agreement with the following statements: 1) “During the past 12 months, I have been hurt by how people reacted to learning I have HIV;” 2) “During the past 12 months, I have stopped socializing with some people because of their reactions to my HIV status;” 3) “During the past 12 months, I have lost friends by telling them I have HIV;” 4) “I am very careful who I tell that I have HIV;” 5) “I worry that people who know I have HIV will tell others;” 6) “I feel that I am not as good a person as others because I have HIV;” 7) “Having HIV makes me feel unclean;” 8) “Having HIV makes me feel that I’m a bad person;” 9) “Most people think that a person with HIV is disgusting;” and 10) “Most people with HIV are rejected when others find out.” Median scores and 95% CIs were calculated on a scale of 0 (no stigma) to 100 (highest stigma).

§§
https://www.cdc.gov/hiv/research/interventionresearch/compendium/index.html

¶¶
https://together.takemehome.org

***
https://www.cdc.gov/hiv/funding/announcements/ps20-2010/index.html
